# Design of a Broad-Range Bacteriophage Cocktail That Reduces Pseudomonas aeruginosa Biofilms and Treats Acute Infections in Two Animal Models

**DOI:** 10.1128/AAC.02573-17

**Published:** 2018-05-25

**Authors:** Francesca Forti, Dwayne R. Roach, Marco Cafora, Maria E. Pasini, David S. Horner, Ersilia V. Fiscarelli, Martina Rossitto, Lisa Cariani, Federica Briani, Laurent Debarbieux, Daniela Ghisotti

**Affiliations:** a Department of Biosciences, University of Milan, Milan, Italy; b Department of Microbiology, Institut Pasteur, Paris, France; c Children's Hospital Bambino Gesù IRCCS, Rome, Italy; d Fondazione IRCCS Ca' Granda, Ospedale Maggiore Policlinico, University of Milan, Milan, Italy

**Keywords:** Galleria mellonella, Pseudomonas aeruginosa, bacteriophages, cystic fibrosis, phage therapy

## Abstract

The alarming diffusion of multidrug-resistant (MDR) bacterial strains requires investigations on nonantibiotic therapies. Among such therapies, the use of bacteriophages (phages) as antimicrobial agents, namely, phage therapy, is a promising treatment strategy supported by the findings of recent successful compassionate treatments in Europe and the United States. In this work, we combined host range and genomic information to design a 6-phage cocktail killing several clinical strains of Pseudomonas aeruginosa, including those collected from Italian cystic fibrosis (CF) patients, and analyzed the cocktail performance. We demonstrated that the cocktail composed of four novel phages (PYO2, DEV, E215 and E217) and two previously characterized phages (PAK_P1 and PAK_P4) was able to lyse P. aeruginosa both in planktonic liquid cultures and in biofilms. In addition, we showed that the phage cocktail could cure acute respiratory infection in mice and treat bacteremia in wax moth (Galleria mellonella) larvae. Furthermore, administration of the cocktail to larvae prior to bacterial infection provided prophylaxis. In this regard, the efficiency of the phage cocktail was found to be unaffected by the MDR or mucoid phenotype of the pseudomonal strain. The cocktail was found to be superior to the individual phages in destroying biofilms and providing a faster treatment in mice. We also found the Galleria larva model to be cost-effective for testing the susceptibility of clinical strains to phages, suggesting that it could be implemented in the frame of developing personalized phage therapies.

## INTRODUCTION

The opportunistic pathogen Pseudomonas aeruginosa principally infects the airways of immunocompromised patients and is one of the principal bacteria isolated from adults with cystic fibrosis (CF). The appearance and diffusion of multidrug-resistant (MDR) isolates of P. aeruginosa are responsible for the increasingly unsuccessful use of antibiotics. Thus, alternative therapies are urgently needed, and the use of bacteriophages (phages), the natural viral enemies of bacteria, has received renewed attention (1, 2). Phage therapy was proposed 100 years ago, before the discovery of antibiotics (3). Following an initial worldwide expansion, the use of this therapy declined, being replaced by the use of antibiotics, which were more successful. Beyond efficacy itself, the lack of precise knowledge of the complex interaction between phages and bacteria has played a major role in the shift from using phages to using antibiotics. For instance, while today genome sequencing and experimentation can determine whether a phage is virulent or temperate, such information was not available in early 20th century. Temperate phages are known to serve as vehicles for bacterial sequence exchanges between strains, eventually leading to the dissemination of genes coding for toxins or antibiotic resistance. Therefore, for therapeutic applications, the use of only virulent (strictly lytic) phages is advised. Compared to antibiotics, phages have several advantages. First, as obligate bacterial viruses, they tend to be specific to their bacterial hosts and their killing activity is confined to a narrow range of pathogenic strains. This avoids collateral damage to human and animal healthy commensal microbiota, contrary to broad-spectrum antibiotics (4, 5). Another advantage of phage therapy over conventional antibiotics is the dynamic dosing provided by phages, which multiply when the target bacterial host strains are present and decrease in number as the target bacteria are eliminated (6). Thus, phages provide an infection site-specific augmentation of the dose that cannot be achieved through the standard repeated dosing of antibiotics. Lastly, phages are often able to kill bacteria independently of their MDR phenotype (7, 8).

Several reports have demonstrated that the growth of a pathogenic bacterium can be controlled *in vitro* and *in vivo* with specific phages. These range from the experimental treatment of Escherichia coli diarrhea (9), Klebsiella pneumoniae pulmonary infection (10), and Acinetobacter baumannii pneumonia (11) to that of P. aeruginosa keratitis (12). In addition, the therapeutic effect of phage administration on P. aeruginosa-infected mice (13–15) or Galleria larvae has also been reported (16). However, no consensual and validated guidelines for the selection of individual or multiple therapeutic phages that target a specific pathogen have been adopted (17). One strategy is to isolate the causative agent of the bacterial infection from the patient and then identify *in vitro* one or more phages that lyse that strain(s). This approach is laborious and time-consuming, and it also requires a large pool of phages to be on hand. An alternative strategy would be to preemptively combine a mixture of phages (cocktail) that together are able to efficiently kill a broad range of clinical strains of a disease-specific pathogen (18).

In this study, we investigated the preemptive tactic, using *in vitro* and *in silico* criteria to combine a mixture of phages to efficiently treat a broad range of P. aeruginosa clinical strains isolated from Italian patients with CF. We show that a cocktail of six phages virulent for Pseudomonas can kill with 77% coverage P. aeruginosa clinical strains from CF patients, while it is effective at reducing pseudomonal biofilms *in vitro*. Furthermore, the phage cocktail resolved pseudomonal acute pneumonia in mice and treated bacteremia in wax moth (Galleria mellonella) larvae.

## RESULTS

### Isolation and electron microscopy imaging of broad-host-range phages infecting Italian P. aeruginosa strains isolated from cystic fibrosis patients.

We collected 40 P. aeruginosa strains from CF patients at several Italian medical centers (see Materials and Methods). In addition, we added 2 Italian isolates from patients with chronic obstructive pulmonary disease, 9 non-Italian clinical strains previously isolated from CF and non-CF patients, 5 environmental isolates, and 2 laboratory strains (PAO1 and PAO1 *pilA*) to increase the genetic diversity of the bacterial hosts tested (see Table S1 in the supplemental material).

Then, we isolated 23 novel phages as described in Materials and Methods (Table S2). Next, we measured the efficiency of plating (EOP) of each phage on the panel of 58 P. aeruginosa strains (Table S3). As expected, each phage had a distinct host range, with no individual phage being able to lyse all strains in the aforementioned collection, and some only lysed less than 30% of strains. Intriguingly, none of our tested phages was able to lyse P. aeruginosa strains PaPh23 and PaPh30. However, about half of the isolated phages exhibited a much broader host range by lysing more than half of the strain collection.

From the pool of moderately broad host range phages, we selected six phages (PYO2, E215, E220, S218, E217, and DEV) that, when combined, were theoretically predicted to infect 97% of the strain collection (Table S3). Transmission electron microscopy images of these phages are shown in Fig. S1. All belong to the order Caudovirales; PYO2, DEV and E220 are members of the Podoviridae family and share a highly similar morphology (a regular icosahedral head of 72 nm in diameter and a short tail of 18 nm); E215 and E217 belong to the Myoviridae family, and both share a very similar morphology with a regular icosahedral head of 80 nm in diameter and a tail 185 nm long; S218 is a member of the Siphoviridae and possesses an icosahedral head of 100 nm in length and 60 nm in width and a flexible 210-nm-long tail.

One-step growth experiments in PAO1 were performed in order to characterize the PYO2, DEV, E215, and E217 phages (Fig. S2). Phages PYO2 and DEV have a similar latent period (20 min) and burst size (100 and 200 PFU/ml, respectively), whereas E215 and E217 show a longer latent period (30 to 40 min), and both have a burst size of over 200 PFU/ml.

### Genome characterization of the selected phages.

We sequenced phages PYO2, E215, E220, S218, E217, and DEV, and their genomic characteristics are reported in Table S4. The genomes of both phages PYO2 and DEV were 72,697 bp in length and 99% similar. The alignments showed that the sequences of PYO2 and DEV are related to those of the Podoviridae *Lit1virus* group and share similar levels of conservation with the publically available sequences of Pseudomonas phages PEV2 and RWG. The genomes of phages E215 (66,789 bp) and E217 (66,291 bp) also had a high level of similarity (97% identity over 98% of their length), are related to the genomes of members of the *P1virus* subfamily of the Myoviridae, and closely resemble the genome of Pseudomonas phage vB_PaeM_CEB_DP1.

In order to discard temperate phages and, more broadly, phages that could serve as vehicles for undesirable functions, we searched for similarities between all putative viral open reading frames (ORFs) and a custom database of genes expressing bacterial virulence factors, antibiotic resistance, and integrases/excisionases/recombinases. We found that the genomes of phages PYO2, DEV, E215, and E217 do not encode proteins with similarity to undesirable functions, leading us to classify them as virulent. On the contrary, as reported in Table S4, the genomes of phages E220 and S218 contained open reading frames with high levels of similarity to genes annotated as integrases in the ACLAME database (19). This suggests that phages E220 and S218 have a temperate life cycle not conducive of a desirable antimicrobial agent and thus were not studied further.

### Evaluation of *in vitro* efficiency of transduction of the selected phages.

We tested the capacity of the PYO2, DEV, E215, and E217 phages to transduce genetic markers. We found that the frequency of transduction was less than 10^−9^ per infecting phage, a frequency that is about 100-fold lower than the typical general transduction rate for virulent phages, leading us to conclude that these phages would unlikely serve as vehicles to carry antibiotic resistance or virulence genes (20).

### Definition of a genetically diverse phage cocktail.

With the aim to assemble a phage cocktail that displays a broad host range and genetic diversity, we selected 6 virulent phages, PYO2, DEV, E215, E217, PAK_P1, and PAK_P4. The last two are previously characterized P. aeruginosa Myoviridae virulent phages isolated in France (13, 21, 22) that are 93,198 bp and 93,147 bp, respectively, and that display 93% identity over 98% of their genome length. PYO2/DEV, E215/E217, and PAK_P1/PAK_P4 constitute three groups of genomically similar phages and represent three viral genera. The six phages do not show significant sequence similarity to each other, other than the sequence similarity between the pairs of closely related phages (Fig. S4 and Table S4). Here, we refer to the mixture of all six phages as the “phage cocktail.”

### *In vitro* characterization of the cocktail and its individual components.

The EOP of the cocktail was compared to that of each individual phage on 58 P. aeruginosa strains and is reported in Table 1. As was to be expected, the phage cocktail was able to lyse a range of bacterial strains broader than the range that any of the individual phages that made up the cocktail could lyse. That is, a single phage with the broadest host range lysed only 36 of the 58 strains in the full strain collection (62%) and 22 of the 40 Italian CF clinical strains (55%), whereas combining the phages in a cocktail expanded the host range of the full strain collection lysed by 15% (45 out of 58 strains) and the host range of the Italian CF clinical strain collection lysed by 20% (30 out of 40 strains). Of note, the host range of the phage cocktail *in vitro* was lower than that predicted theoretically from summing the host range of each individual phage within the cocktail. This phenomenon could be due to host infection competition between phages (23, 24).

**TABLE 1:**
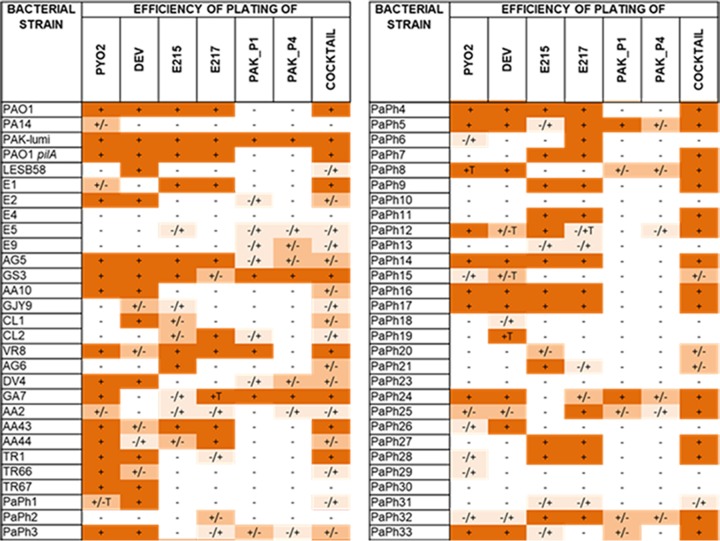
Efficiency of plating of single phages selected for the cocktail and of the cocktail^*a*^

a Five microliters of 10-fold serial dilutions of the indicated single phages or of the phage cocktail was spotted on a lawn of each specific bacterial host; the plates were observed after overnight incubation at 37°C. +, EOP of 1; +/−, EOP of 10^−1^ to 10^−2^; −/+, EOP of 10^−3^; −, EOP of <10^−4^; T, turbid plaques.

**FIG 1:**
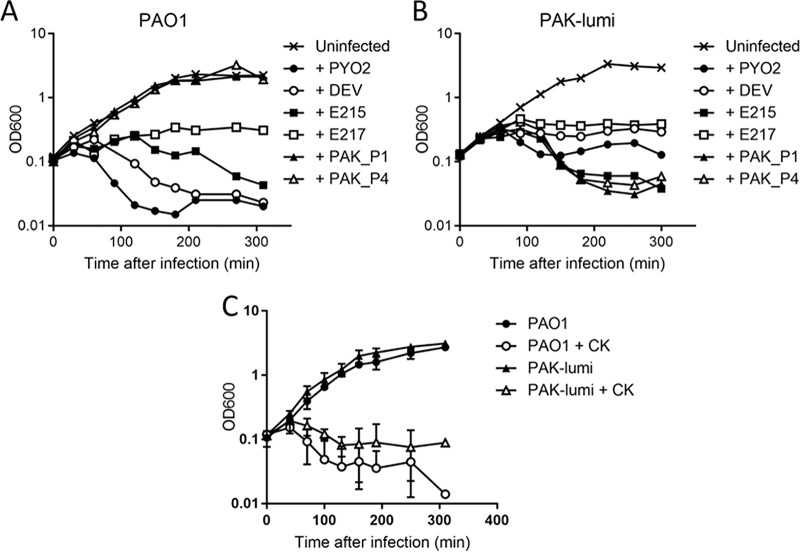
Growth kinetics of P. aeruginosa cells in liquid culture in the presence of phages. (A and B) Exponentially growing bacteria (OD_600_ = 0.1) of either strain PAO1 (A) or strain PAK-lumi (B) were infected by the indicated phages, each at an MOI of 2.5. For clarity, the results of only one out of three independent experiments are shown. The infection with E217 was repeated three times with each strain, with superimposable results. (C) PAO1 and PAK-lumi infection with the 6-phage cocktail (CK; total MOI = 2.5). The average and SD from two independent experiments are shown.

The lysis kinetics of P. aeruginosa strain PAO1 and PAK-lumi cultures infected with each phage and with the cocktail were followed by monitoring the optical density (OD) at 600 nm (OD_600_) over time (Fig. 1A to C). For PAO1, at a multiplicity of infection (MOI) of 2.5, PYO2 and DEV caused a decrease in the OD_600_ at 1 to 1.5 h postinfection (p.i.) and E215 caused a relatively smaller decrease at about 2 h. For PAK-lumi, the effect was less pronounced both after DEV infection and after E215 infection. Interestingly, phage E217 did not cause lysis but clearly stopped the growth of both PAO1 and PAK-lumi. PAK_P1 and PAK_P4 did not alter PAO1 growth, whereas the OD_600_ of PAK-lumi started to decrease at 2 h p.i.

**FIG 2:**
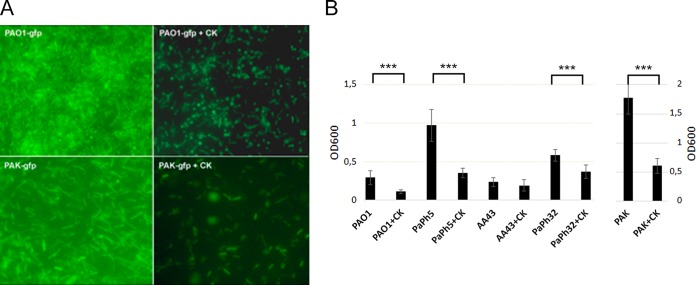
Disruption of the P. aeruginosa biofilm by the phage cocktail. (A) Forty-eight-hour biofilms of PAO1 *gfp* and PAK *gfp* without and after addition of the 6-phage cocktail (+CK). (B) Twenty-four-hour biofilms of the indicated P. aeruginosa strains were exposed for 4 h to the phage cocktail. The reduction of the biofilm biomass following treatment with the phage cocktail was compared with that following no treatment by measuring the OD_600_ after crystal violet staining. The biofilm reduction by the cocktail reached 62% for PAO1, 64% for PaPh5, 19% for AA43 (*P* = 0.6), 37% for PaPh32, and 66% for PAK-lumi. The error bars indicate standard deviations, and the statistical significance of the biofilm reduction (***, *P* < 0.001) was assessed by Student's *t* test.

After infection of both the PAO1 and PAK-lumi cultures with the phage cocktail (Fig. 2C), the OD_600_ decreased at about 70 min p.i. This indicates that the phages were able to kill sensitive bacteria *in vitro* in a relatively short time after infection. After overnight incubation, however, the OD_600_ reached high values, due to the growth of resistant bacteria, as confirmed by testing several bacteria (10/10 resistant clones), indicating that the cocktail failed to prevent the outgrowth of phage-resistant variants.

### Phages disrupt P. aeruginosa biofilm.

We tested the capability of phages to reduce the biofilms formed by green fluorescent protein (GFP)-expressing P. aeruginosa PAO1 or PAK-lumi strains. After 48 h of biofilm formation on glass slides, the phages were applied as a cocktail (Fig. 2A) or individually (Fig. S3). The biofilm biomass nearly disappeared after incubation with the phage cocktail. To quantify the biofilm reduction, we measured the biofilm biomass by crystal violet staining of 24-h biofilms and found that the cocktail caused a significant reduction in the PAO1 and PAK-lumi biofilm biomass (63% and 65%, respectively; Fig. 2B). In addition, the efficiency of the cocktail in destroying a preformed biofilm formed by various clinical P. aeruginosa strains was also tested. The cocktail reduced 64% (*P* < 0.001) of a highly dense biofilm produced by strain PaPh5 used in the first infection and reduced the biomasses of the mucoid AA43 strain and the mucoid MDR PaPh32 strain by 19% (*P* = 0.6) and 37% (*P* < 0.001), respectively.

Intriguingly, PAK_P1 and PAK_P4, which are unable to replicate on PAO1, caused a significant increase in the PAO1 biofilm biomass (Fig. S3). This effect was not observed when the phages were components of the cocktail. Although the cause of this phenomenon is unclear, it could be related to the host defense system to reduce phage growth (25).

### Phage treatment of P. aeruginosa respiratory infection in mice.

**FIG 3:**
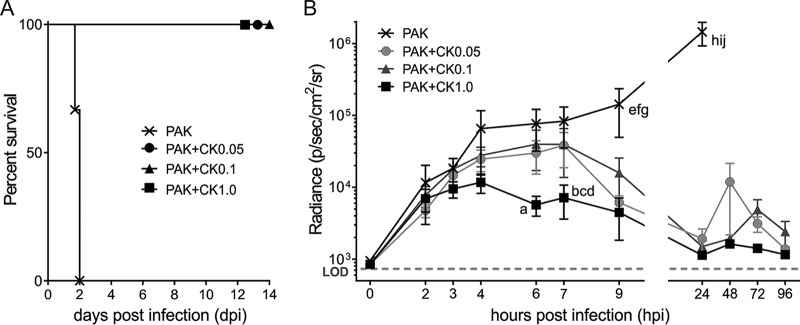
The phage cocktail fully cures acute respiratory infections in mice. (A) Fatal respiratory infections after 1 × 10^7^ CFU of P. aeruginosa strain PAK-lumi (PAK) (*n* = 5 mice for each treatment group and *n* = 3 for untreated mice) was intranasally instilled into mice were cured by administration of the 6-phage cocktail (CK) at 2 h postinfection. The cocktail consisted of a mixture of six Pseudomonas phages each given at an MOI of 0.05, 0.1, or 1.0 (CK0.05, CK0.1, and CK1.0, respectively). (B) Photon emission of the chest area of infected mice quantified using an IVIS Spectrum 100 imaging system (p/sec/cm^2^/sr, number of photons per second per square centimeter per steradian). The letters beside the data points indicate significance by two-way ANOVA with the Tukey correction, as follows: a, *P* = 0.0012 for PAK versus PAK+CK1.0; b, *P* < 0.0001 for PAK versus PAK+CK1.0; c, *P* = 0.0406 for PAK+CK0.05 versus PAK+CK1.0; d, *P* = 0.0365 for PAK+CK0.1 versus PAK+CK1.0; e, *P* = 0.0003 for PAK versus PAK+CK0.05; f, *P* = 0.0008 for PAK versus PAK+CK0.1; g, *P* < 0.0001 for PAK versus PAK+CK1.0; h, *P* < 0.0001 for PAK versus PAK+CK0.05; i, *P* < 0.0001 for PAK versus PAK+CK0.1; and j, *P* < 0.0001 for PAK versus PAK+CK1.0.

We tested the capacity of the phage cocktail to cure a P. aeruginosa acute respiratory infection in a mouse model. Figure 3 shows that phage treatment even at the lowest MOI (an MOI of 0.05 for each phage) was effective at reducing the respiratory bacterial burden by 48 h and achieving a 100% survival rate. Noninvasive longitudinal monitoring of P. aeruginosa infection showed that the phage cocktail administered at each of the MOIs tested led to a reduction of the bacterial burden in the mouse lungs (Fig. 3B). In comparison, administration of the phage cocktail at the highest MOI (1.0) began to reduce significantly the bacterial density by 6 h posttreatment, whereas with administration at the two lower MOIs (0.05 and 0.1), it took up to 9 h before the reduction became significant.

### Phage treatment of P. aeruginosa systemic infection of G. mellonella larvae.

**FIG 4:**
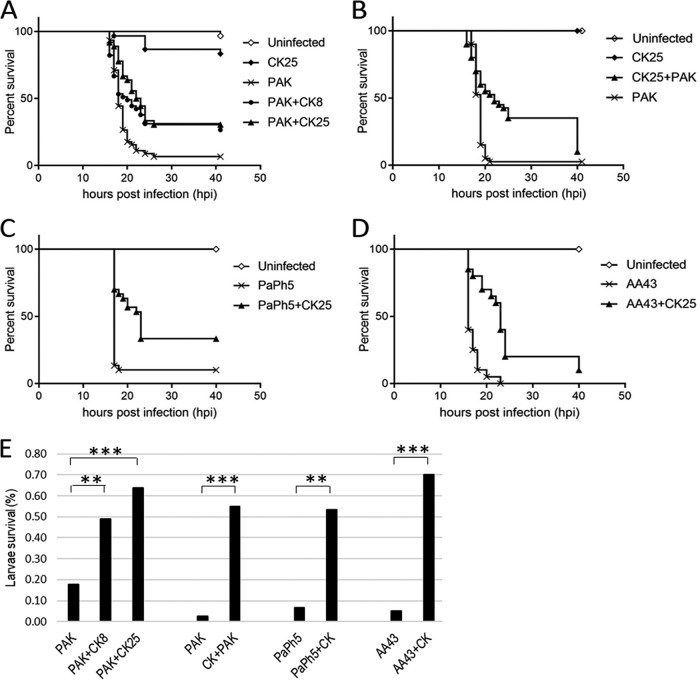
The phage cocktail prolongs the survival of systemically infected G. mellonella larvae. (A) Kaplan-Meier survival curves of larvae infected with PAK-lumi (30 CFU/larva, *n* = 45 per group) and treated 1 h after infection with PBS (PAK) or the 6-phage cocktail (CK) at two different MOIs of 8 (PAK+CK8) and 25 (PAK+CK25). In addition, uninfected larvae received the cocktail at an MOI of 25 (CK25). Pairwise comparisons between untreated infected larvae and phage-treated infected larvae using the Mantel-Cox test indicated a significant difference (*P* < 0.0001 for both CK8 and CK25). (B) Kaplan-Meier survival curves of larvae prophylactically treated at an MOI of 25 1 h before PAK-lumi challenge (CK25+PAK) or PBS challenge (CK25) (*n* = 20 per group). Pairwise comparisons between untreated infected larvae and phage-treated infected larvae using the Mantel-Cox test indicated a significant difference (*P* < 0.0001). (C and D) Kaplan-Meier survival curves of larvae infected with PaPh5 (30 CFU/larva) or AA43 (110 CFU/larva) (*n* = 35 per group) and treated 1 h later with PBS (PaPh5 and AA43) or the 6-phage cocktail (PaPh5+CK25 and AA43+CK25). Pairwise comparisons between untreated infected larvae and phage-treated infected larvae using the Mantel-Cox test indicated a significant difference (for PaPh5, *P* < 0.0001; for AA43, *P* < 0.0001). (E) Comparison of the rates of survival of larvae treated with the phage cocktail at 20 h after infection with the indicated strains. Statistical significance was assessed by the chi-square test with the Yates correction when needed: PAK versus PAK+CK8, *P* < 0.01 (**); PAK versus PAK+CK25, *P* < 0.0001 (***); PAK versus CK+PAK, *P* < 0.0001 (***); PaPh5 versus PaPh5+CK, *P* < 0.01 (**); and AA43 versus AA43+CK, *P* < 0.0001 (***).

In the frame of developing personalized phage therapies before the treatment of patients, we investigated the effects of the phages on P. aeruginosa clinical isolates in wax moth (G. mellonella) larvae as a rapid and economical screening method. First, we assessed that administration of the phage cocktail at the higher dose of an MOI of 25 (CK25) did not *per se* cause adverse effects on the larvae (Fig. 4A and B). Then, we showed that death was significantly delayed in larvae infected with a lethal dose of PAK-lumi and treated 1 h later with the phage cocktail (Fig. 4A). Indeed, the rate of survival of the larvae after 20 h increased from about 17% to 49% by treatment with the cocktail at an MOI of 8 (CK8) and to 63% by treatment with the cocktail at an MOI of 25 (CK25) (Fig. 4E). Even at a later time point (40 h), the rate of survival increased from 6.6% of larvae not treated with phages to 26.6% and 30.5% (Mantel-Cox test, *P* < 0.0001) of the groups that received the phage cocktail at the different MOIs (Fig. 4A). Moreover, we showed that pretreatment with the phage cocktail 1 h before PAK-lumi challenge provided prophylaxis against lethal infection (Mantel-Cox test, *P* < 0.0001) (Fig. 4B and E). Interestingly, both of the two clinical strains PaPh5 and AA43 were also controlled by the phage cocktail (*P* < 0.0001) (Fig. 4C to E).

## DISCUSSION

CF patients, who experience pulmonary infections predominantly caused by P. aeruginosa, are increasingly exposed to the risk of infection caused by MDR strains due to the recurrent use of antibiotics. In this study, we isolated and characterized new phages, assembled a 6-phage cocktail, and tested its efficacy against MDR P. aeruginosa strains both *in vitro* and in two *in vivo* animal models.

Several studies have reported the successful treatment of experimental bacterial infections with phages, supporting their use as first-line therapy, in particular, for infections caused by MDR pathogens (26–29). In line with these data, two compassionate phage treatments were recently reported in Europe and the United States, confirming the efficacy and safety of such an approach (30, 31). However, for these two examples, the choice of phages was guided, first, by their *in vitro* activity against the patient's pathogen without, to our knowledge, any *in vivo* validation step. In addition to such a customized solution, a parallel strategy would be to design ready-to-use cocktails with a broad host range. Here, we assembled a 6-phage cocktail, taking into account the host range and genomic information for phages, and assessed its *in vitro* and *in vivo* efficacies.

The cocktail is made up of 3 pairs of closely related phages, as revealed by genome sequencing (with divergences of less than 8% sequence identity between the components of each pair). Although the pairs have highly similar genomes, the related phages differ in their host range *in vitro*, which is part of the rationale for the construction of the cocktail. For example, phage DEV lyses strain LESB58, whereas the closely related phage PYO2 does not. The nucleotide sequences of these two phages, however, differ by less than 1% over their complete genomes. We noticed that the genomic variation is mainly confined to two genes: the 3′-end region of the RNA polymerase gene (*orf71* in Fig. S4B in the supplemental material), in which most substitutions observed were synonymous, and the ORF encoding dUTPase (*orf81* in Fig. S4B), in which 21/128 amino acids (16%) differed. dUTPase has been reported to be essential for viral replication in certain hosts (32) and implicated in the host range under specific conditions (33). Our observations suggest that dUTPase genes can be considered attractive candidate genes, beyond the expected structural genes involved in host recognition, for future studies of host specificity mechanisms (34).

Compared to the host spectrum of the individual phages, our cocktail had *in vitro* a broader host spectrum for clinically isolated P. aeruginosa strains. In addition, the cocktail lysed all 3 MDR strains in our collection (strains PaPh24, PaPh25, and PaPh32), implying that phage infection was independent of cells harboring an antibiotic resistance mechanism. Moreover, the phage cocktail was able to infect and kill mucoid strains isolated from chronically infected CF patients.

Treatment of a P. aeruginosa biofilm with the phage cocktail demonstrated that phages are able to enter the biofilm, destroying the biomass and reaching the bacteria embedded inside. In this respect, the use of the phage cocktail greatly increased the effect of single-phage infections (compare Fig. 2 with Fig. S3). We also observed that the phage cocktail reduced to different degrees the biofilms formed by different strains, which may have been due to the differences in biofilm formation and the biofilm composition observed with clinical P. aeruginosa strains (35).

Our findings indicate that in mice, lethal acute respiratory infection can be cured by treatment with the cocktail. Compared to our previous data obtained with a single phage, the cocktail showed the advantage of having more rapid efficacy in reducing the bacterial load (13, 21). This suggests a synergistic action when using multiple phages. Further investigations will be required to identify the mechanisms behind such synergy, but in the light of the findings of our recent investigations on the role of the immune system during monophage therapy, we can hypothesize that the cocktail reduces the probability that phage-resistant bacteria will grow (15). In systemic G. mellonella infection, in which bacteria were directly injected in the hemolymph, a significant delay in the time to death over that for the nontreated controls was observed upon phage injection. The presence of a significant difference in lethality between untreated and phage-treated larvae at an early time point after infection (20 h) suggests that this test could be introduced for *in vivo* evaluation of the effectiveness of phage therapy. Moreover, the phage cocktail was able to prevent P. aeruginosa infection in the larvae. Prophylaxis with phages could be proposed for CF or immunocompromised patients, who are frequently hospitalized and therefore at higher risk of exposure to nosocomial infections. The use of the larva model provided several advantages over the use of the murine model, among which was the flexibility to test many clinical strains, as shown here. Indeed, some P. aeruginosa clinical strains have been found not to infect the respiratory tract of mice (36; unpublished data), which limits the use of this model. Other advantages are related to the cost, the easy management in a microbiology lab, and to some extent, the experimental time. These characteristics of the larva model provide a solution for the *in vivo* evaluation of the activities of phages and cocktails against a clinical isolate that could be integrated into the process of selection of the phages best suited for use in the formulation of a cocktail.

Overall, our strategy, based on (i) host range, (ii) genomic information, and (iii) *in vitro* efficacies, led to the formulation of a 6-phage cocktail that was validated in two *in vivo* models. It should be noticed that the *in vitro* efficacies in liquid cultures and biofilms were the less encouraging data, as the cocktail did not prevent within 24 h the growth of bacterial resistant clones, and some individual phages enhanced the density of biofilms. Therefore, to design phage cocktails, the pertinence of *in vitro* tests under conditions irrelevant to the treatment of human bacterial infections can be questioned, despite its rationale being at the root of phage therapy (37).

## MATERIALS AND METHODS

### P. aeruginosa strains.

Clinical isolates of P. aeruginosa were isolated from primary and chronically infected patients at the Centro di Riferimento per la Fibrosi Cistica della Regione Lombardia, Milan, Italy, and at the Ospedale Bambino Gesù, Rome, Italy, and kindly provided by A. Bragonzi of the Infection and Cystic Fibrosis Unit at San Raffaele, Milan, Italy. Strain PAO1 *pilA*, in which the *pilA* gene has been deleted, was kindly provided by F. Imperi (Università degli Studi di Roma-Sapienza, Rome, Italy). The other strains were present in the lab collection. All the strains are listed in Table S1 in the supplemental material. Strains PAO1 and PAK-lumi were transformed with plasmid tPUCP19-GFP (C. Penaranda and D. Hung, personal communication), which expresses high levels of GFP to make them fluorescent, and strain PAO1Tc^r^ (38) was used for transduction experiments.

### Bacteriophage isolation.

Our phage collection includes 25 independent isolates, listed in Table S2. Several isolates originated from sewage samples collected from the Milano Nosedo and Milano Peschiera Borromeo wastewater treatment plants. Independent samples collected on different days were used to avoid the reisolation of the same phage. Clear plaques were purified by standard procedures (39). Two phages were isolated from the commercial preparations Phagyo (batch number 06.06.13; JSC Biochimpharm, Tbilisi, Georgia) and Intesti Bacteriophage (batch number M2-501; Eliava Institute, Tbilisi, Georgia) by plating the preparation on PAO1 and isolating a single clear plaque from each preparation. Several natural derivatives of these two initial isolates with different plaque morphologies or host ranges were added to the collection. On a few occasions, the evolution of some phage variants was performed, as indicated previously (40), in which an exponential-phase culture of a specific strain was infected with a phage and infection was continued for 24 h; then, the culture was centrifuged and the supernatant was used to reinoculate a fresh bacterial culture. This procedure was repeated daily for 6 days. The EOP of the evolved phage present after 6 days was compared to the EOP of the ancestral phage used as the inoculum. If the EOP was improved, the phage was added to the collection. Despite several attempts, no phage able to infect strains PaPh23 and PaPh30 was isolated.

### High-titer phage stock preparations.

High-titer preparations of the phages were obtained by infection of 500 ml of liquid culture of PAO1 or PAK-lumi, as described in reference 21, with the following modifications: the lysates were filtered through 1.2-μm-pore-diameter filters and incubated for 30 min at 37°C with DNase (1 μg/ml) and RNase (1 μg/ml) before precipitation with polyethylene glycol. For *in vivo* experiments, phage lysates were purified by cesium chloride ultracentrifugation, as described in reference 41, and dialyzed against TN buffer (10 mM Tris, 150 mM NaCl, pH 7). Then, each phage preparation was passed through an endotoxin removal column (EndoTrap HD; Hyglos, Germany) before measuring the endotoxin level by Limulus amoebocyte lysate chromogenic endotoxin quantitation (Pierce). The levels of endotoxins of the phage preparations were below the limit value recommended for intravenous administration (5.0 international units/kg body mass/hour; http://www.who.int/medicines/publications/pharmacopoeia/Bacterial-endotoxins_QAS11-452_FINAL_July12.pdf).

### Plating efficiency.

The plating efficiency of the isolated phages on clinical strains of P. aeruginosa was determined according to standard protocols: 5 μl of serial dilutions of a phage preparation was spotted on agar plates on which a specific bacterial host was spread. The number of plaques observed after overnight incubation was compared to that obtained on strain PAO1 or PAK-lumi.

### *In vitro* infection.

For determining the lysis kinetics of infected cultures, a culture of strain PAO1 or PAK-lumi in LD broth (42) at 37°C with shaking was infected with each phage at an OD_600_ of 0.1 or with the 6-phage cocktail at an MOI of 2.5, and the OD of the culture was followed.

### Sequencing and assembly of phage genome sequences and their screening for the presence of potential undesirable gene products.

Genomic DNA extracted from purified high-titer phage preparations was subjected to standard Illumina library preparation protocols and sequenced on an Illumina Mi-Seq instrument at the CNR IBBIOM Institute in Bari, Italy, to generate paired-end (2 × 250 nucleotides) sequence reads. Raw paired-end sequence data were subjected to stringent quality trimming and removal of library adapters using Trimmomatic software (43) and assembled using the SPAdes (v3.7.1) program (44) and k-mer lengths of 75, 97, and 119.

Each genome was assembled as a single contig and deposited in GenBank. BLAST similarity searches of the complete genome sequences against the sequences in GenBank recovered high levels of identity and contiguity with previously sequenced genomes and allowed taxonomic assignment of each isolate (Table S4). An in-house database of undesirable genes was constructed by merging entries from the ACLAME database of mobile elements (19) whose descriptions contained any of the terms “integrase,” “excisionase,” “recombinase,” or “repressor” with those from the Comprehensive Antibiotic Resistance Database (CARD) (45) and the Virulence Factor Database (VFDB) (46). All ORFs with the ATG, GTG, or TTG start codon were inferred from viral genome sequences using a custom Python script and used as queries for BLASTX (47) searches (E value cutoff, 5e−04) against the undesirable gene database.

### Transduction assay.

Phages were tested for their ability to transduce the tetracycline resistance (Tc^r^) marker from PAO1Tc^r^ into wild-type PAO1. An aliquot (100 μl) of a high-titer lysate (>1 × 10^10^ PFU/ml) obtained in PAO1Tc^R^ was used to infect a 10-ml overnight culture of the recipient strain PAO1. After static incubation at room temperature for 30 min to allow phage adsorption, the tubes were transferred to 37°C for 20 min, the cells were centrifuged, and the pelleted cells were resuspended in 300 μl LB. Aliquots (150 μl) of the cell mixture were spread onto two agar plates containing tetracycline (100 μl/ml). The frequency of transduction was calculated as the ratio of Tc^r^ colony transductants to the adsorbed phage.

### Composition of the phage cocktail.

Four newly isolated Pseudomonas phages that presented different and complementary host ranges were selected as constituents of the phage cocktail. The four newly isolated phages were named, according to a recent proposal for a rational scheme for the nomenclature of viruses (48), vB_PaeP_PYO2, vB_PaeP_DEV, vB_PaeM_E215, and vB_PaeM_E217, abbreviated in this paper PYO2, DEV, E215, and E217, respectively.

To these, phages PAK_P1 and PAK_P4, previously characterized for their therapeutic efficacies in mouse infections (13, 21), were added; both are Myoviridae, have a head diameter of 80 nm and a tail of about 130 nm, and share no homologies with the other 4 phages in the cocktail. Their genome sequences (GenBank accession number NC_015294 and KC862300, respectively) are 93,198 bp and 93,147 bp, respectively, with 93% identity.

Our final cocktail included these 6 phages: PYO2, DEV, E215, E217, PAK_P1, and PAK_P4. The cocktail was composed of phages mixed at the same number of PFU per milliliter, and the cocktail was prepared immediately before each experiment to ensure accurate phage titers.

### Biofilm disruption.

Two methods were used to monitor biofilm disruption: fluorescence microscopy and crystal violet staining. For better visualization of the biofilms, we used either the PAK-lumi or PAO1 strain transformed with plasmid tPUCP19-GFP, which expresses a high level of GFP. Biofilms of PAK *gfp* and PAO1 *gfp* were grown in an 8-well chamber microscope slide (Nunc Lab-Tek chamber slide) for 48 h in 200 μl LD broth (42) at 37°C. Every 24 h the supernatant was gently removed and replaced by fresh LD broth. After 48 h, phages at 1 × 10^8^ PFU/ml were added and incubation at 37°C was continued for 4 h. The supernatant containing the planktonic cells was removed, and the slide was gently washed and examined with a Leica DMRB microscope equipped with standard fluorescence filters using a 100× objective. Images were acquired with a charge-coupled-device video camera (Leica DCF 480).

For biofilm evaluation by crystal violet staining, an overnight culture of either P. aeruginosa PAO1 or a clinical isolate was diluted to an OD_600_ of about 0.02 in LD broth, and 100 μl was inoculated into 96-well polystyrene microtiter plates. The plates were incubated at 37°C for 24 h to allow biofilm formation. Broth containing planktonic cells was gently removed, the wells were washed with 200 μl of LD, 120 μl of LD containing phage lysate at 10^8^ PFU/ml was added, and incubation was continued for 4 h. After incubation, the wells were carefully emptied and gently washed with H_2_O. The bacteria adhering to the walls of the plate were stained with 150 μl of 0.1% crystal violet solution in H_2_O for 20 min. After washing with tap water, the dye was eluted from the adherent biofilm with 150 μl 5% SDS and quantified by measuring the optical density of a 10-fold dilution of the eluate at 600 nm. Each treatment was repeated in 18 wells, and the median value and standard deviation (SD) were calculated.

### Animals and ethics.

Mice were housed under pathogen-free conditions with *ad libitum* access to food and water. Animal experiments were conducted in accordance with European directives on animal protection and welfare and were approved by the French Ministry of Education and Research (reference number 2015-0041) and the Institut Pasteur (reference number 10.565).

### Mouse acute respiratory infection.

Female BALB/c mice between 8 and 12 weeks of age were anesthetized with 100 mg/kg ketamine and 10 mg/kg xylazine. Subsequently, the animals were intranasally infected with 1 × 10^7^ CFU mid-log-phase P. aeruginosa PAK-lumi suspended in 30 μl phosphate-buffered saline (PBS). After 2 h postinfection (p.i.), mice whose lungs were infected were treated intranasally with the 6-phage cocktail at the indicated PFU dose suspended in 30 μl PBS. An IVIS Spectrum 100 *in vivo* imaging system (PerkinElmer) was used to facilitate noninvasive longitudinal monitoring of P. aeruginosa infection in live individual animals in real time, performed as previously described (13, 49, 50).

### Systemic infection of Galleria mellonella larvae.

A PAK-lumi culture was grown to an OD_600_ of 0.5 in LD broth at 37°C with shaking, pelleted, and diluted to an OD of 1 in physiological solution, equivalent to 1 × 10^9^ CFU/ml. After appropriate dilution, 10 μl of inoculum, containing about 30 cells of P. aeruginosa PAK-lumi, was delivered into the larva hemolymph behind the last proleg. A phage suspension, consisting of 10 μl containing the 6-phage cocktail at 1,500 or 4,500 PFU, was delivered behind the last proleg on the opposite site at 1 h p.i. For prophylaxis experiments, the larvae were infected with the phages 1 h before they were infected with the bacteria. All experiments used 15 or 20 larvae. A positive-control group (larvae infected and treated with physiological solution) and two negative-control groups (one group injected with physiological solution only and one group injected with the phage suspension only to assess the toxicity of the phage cocktail) were also included. The larvae were placed into petri dishes and incubated at 37°C in the dark. The survival of the larvae was followed hourly after 16 h p.i.; the larvae were recorded as dead when they did not move in response to touch.

Phage treatment of larvae infected with clinical P. aeruginosa strains was performed after determination of the lethal dose of bacteria of each strain, equal to 110 and 30 CFU/larva for the AA43 and PaPh5 strains, respectively. At 1 h after injection of the bacteria into the larvae, a fixed dose of phage cocktail (4,500 PFU/larva) was injected.

### Statistical analysis.

Statistical analysis was performed using Student's *t* test or two-way analysis of variance (ANOVA) with the Tukey test or the chi-square test with the Yates correction. *P* values for Kaplan-Meier curves were calculated by the Mantel-Cox test. Statistical analysis was done using GraphPad software (http://www.graphpad.com/quickcalcs/).

### Accession number(s).

The GenBank accession numbers for the phages are MF490236 for vB_PaeP_PYO2, MF490238 for vB_PaeP_DEV, MF490241 for vB_PaeM_E215, MF490240 for vB_PaeM_E217, MF490237 for vB_PaeP_E220, and MF490239 for vB_PaeS_S218.

## Supplementary Material

Supplemental material
