# Supplementary material for: Design of a Broad-Range Bacteriophage Cocktail That Reduces Pseudomonas aeruginosa Biofilms and Treats Acute Infections in Two Animal Models
Source: Antimicrob Agents Chemother. 2018 May 25;62(6):e02573-17. doi: 10.1128/AAC.02573-17 (PMC5971607; doi:10.1128/AAC.02573-17)
Supplement: Supplemental material [file supp_62_6_e02573-17__index.html]

Supplemental material 

# Design of a Broad-Range Bacteriophage Cocktail That Reduces Pseudomonas aeruginosa Biofilms and Treats Acute Infections in Two Animal Models

## Supplemental material

- Supplemental file 1 -

  Supplemental Figures and Tables

  PDF, 2.5M
