# Supplementary material for: Design of a Broad-Range Bacteriophage Cocktail That Reduces Pseudomonas aeruginosa Biofilms and Treats Acute Infections in Two Animal Models
Source: Antimicrob Agents Chemother. 2018 May 25;62(6):e02573-17. doi: 10.1128/AAC.02573-17 (PMC5971607; doi:10.1128/AAC.02573-17)
Supplement: Supplemental material [file AAC.02573-17_zac005187170s1.pdf]

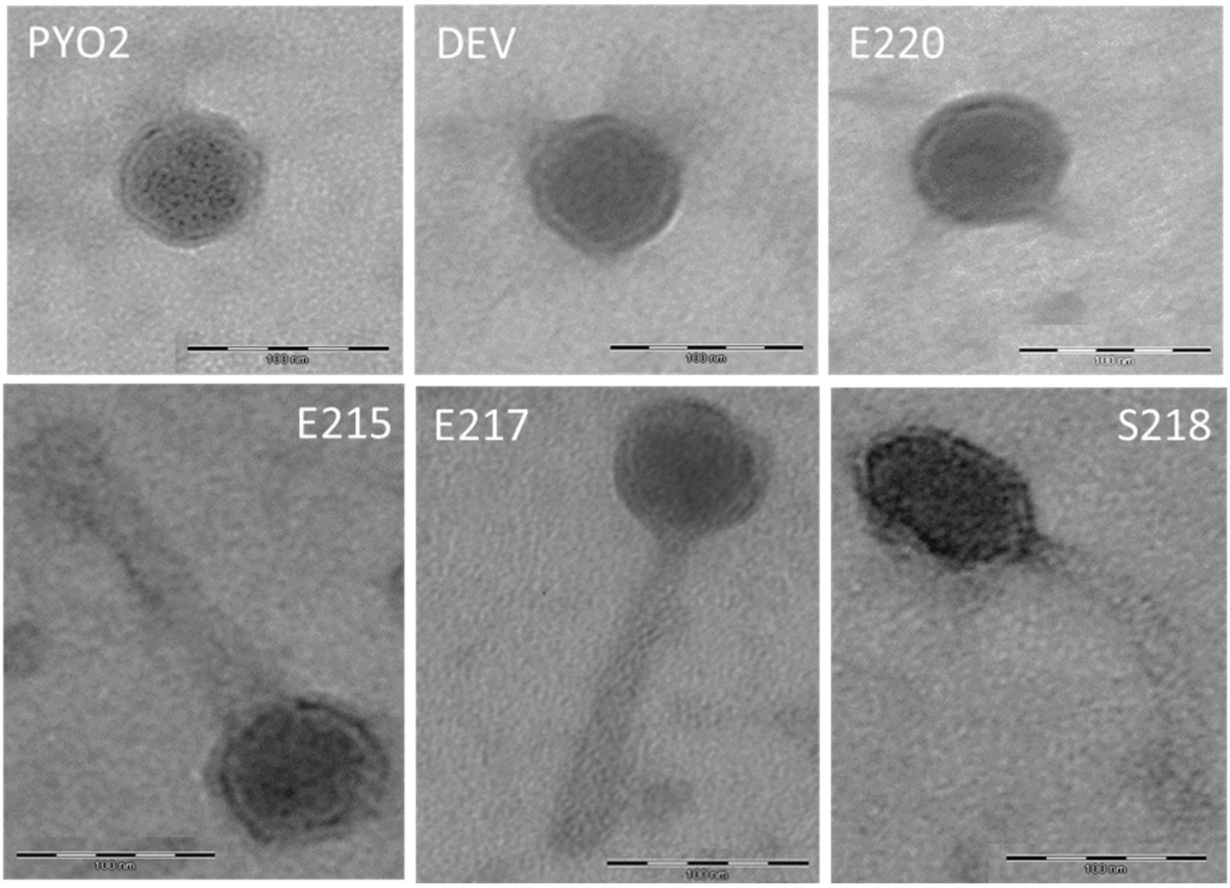

Figure S1. Transmission electron micrograph of the phages

Scale bar, 100 nm. PYO2, DEV and E220 are Podoviridae with regular hycosahedral head of 72 nm diameter and short tail of 18 nm; E215 and E217 are Myoviridae with regular hycosahedral head of 80 nm diameter and tail 185 nm long; S218 is a Siphoviridae, with hycosahedral head of 100 nm in length and 60 nm wide, and a flexible 210 nm long tail.

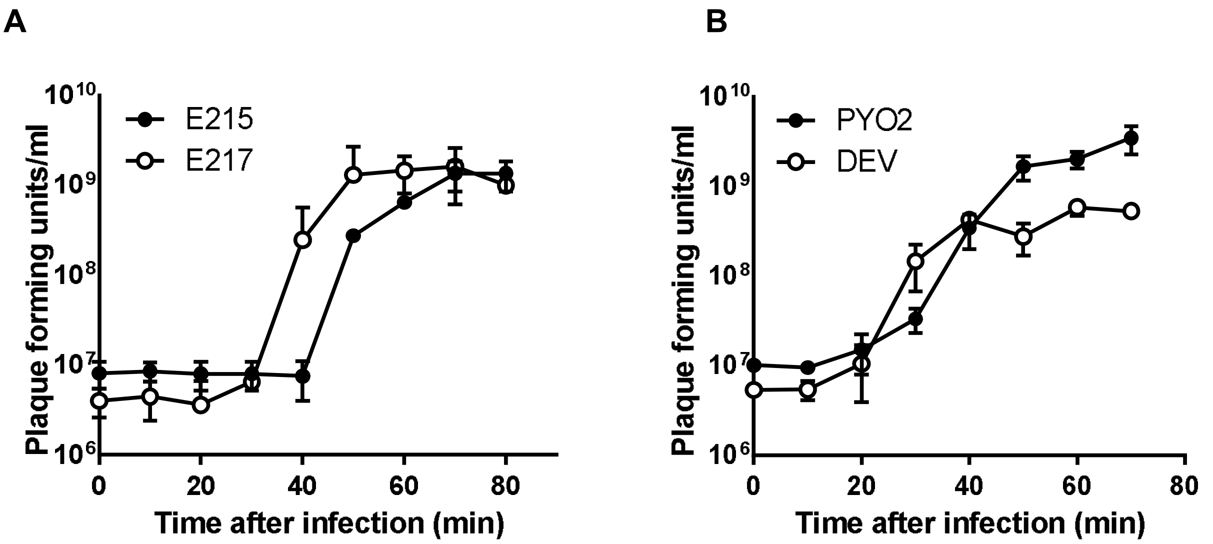

Figure S2. One step growth of *Pseudomonas* phages on strain PAO1. A culture of PAO1 in exponential growth in LD broth at 37°C with shaking was infected with phage at MOI = 0.1, diluted 100-fold in pre-warmed LD broth and samples collected at different times after the infection were plated for determining the plaque forming units/ml (pfu/ml). Latent period and burst size for each infection were calculated. **(A)** PYO2 and DEV. **(B)** E215 and E217. The average and SD of three experiments are reported.

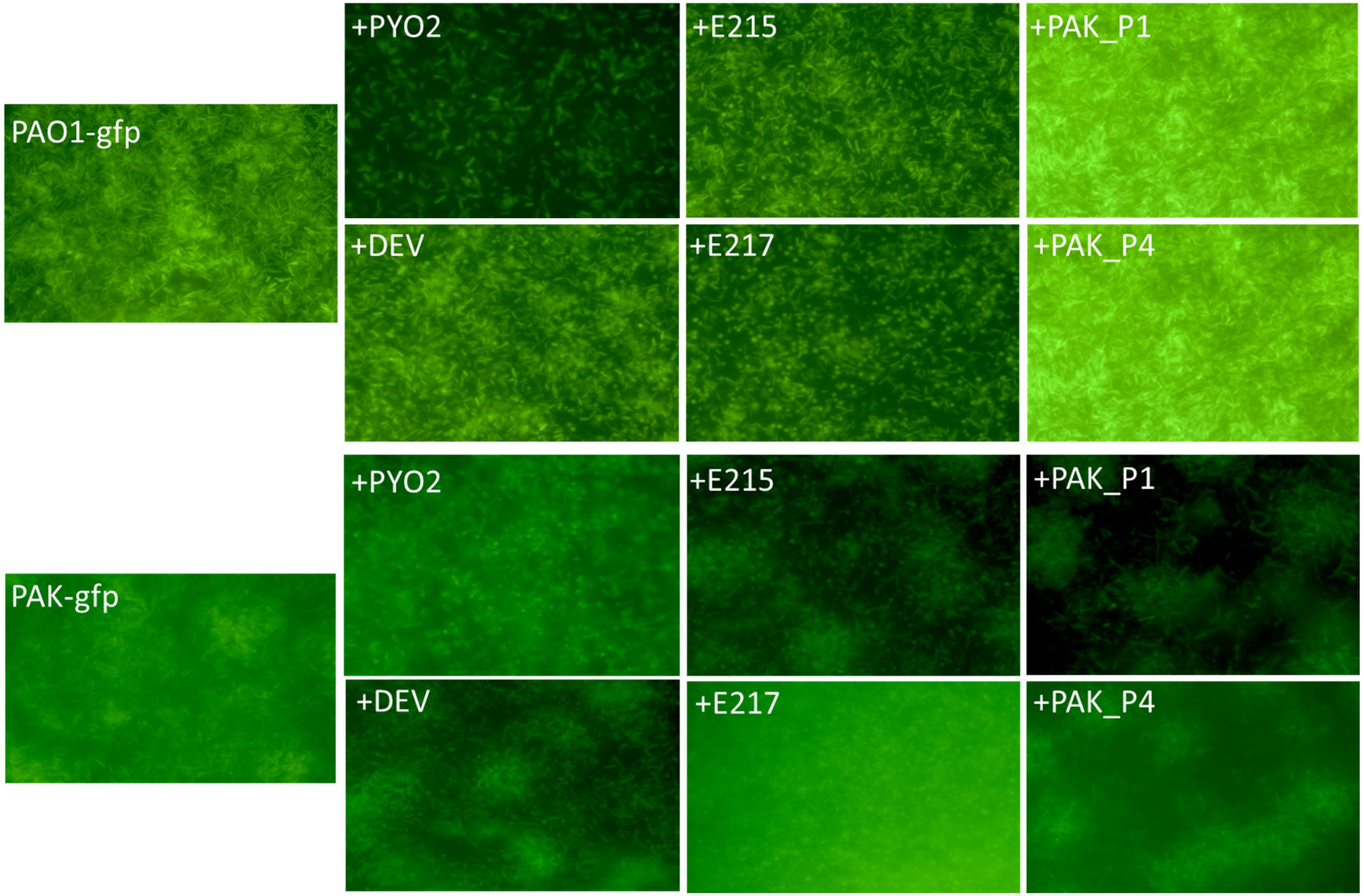

Figure S3. Disruption of the biofilm of PAO1 and PAK by single phage infections. 48 h biofilms of PAO1-gfp and PAK-gfp without and after addition for 4 h of $1x10^8$ pfu of the phage indicated. Observation was performed with a Leica DMRB microscope equipped with standard fluorescence filters using a 100x objective. Images were acquired with a CCD video camera (Leica DCF 480).

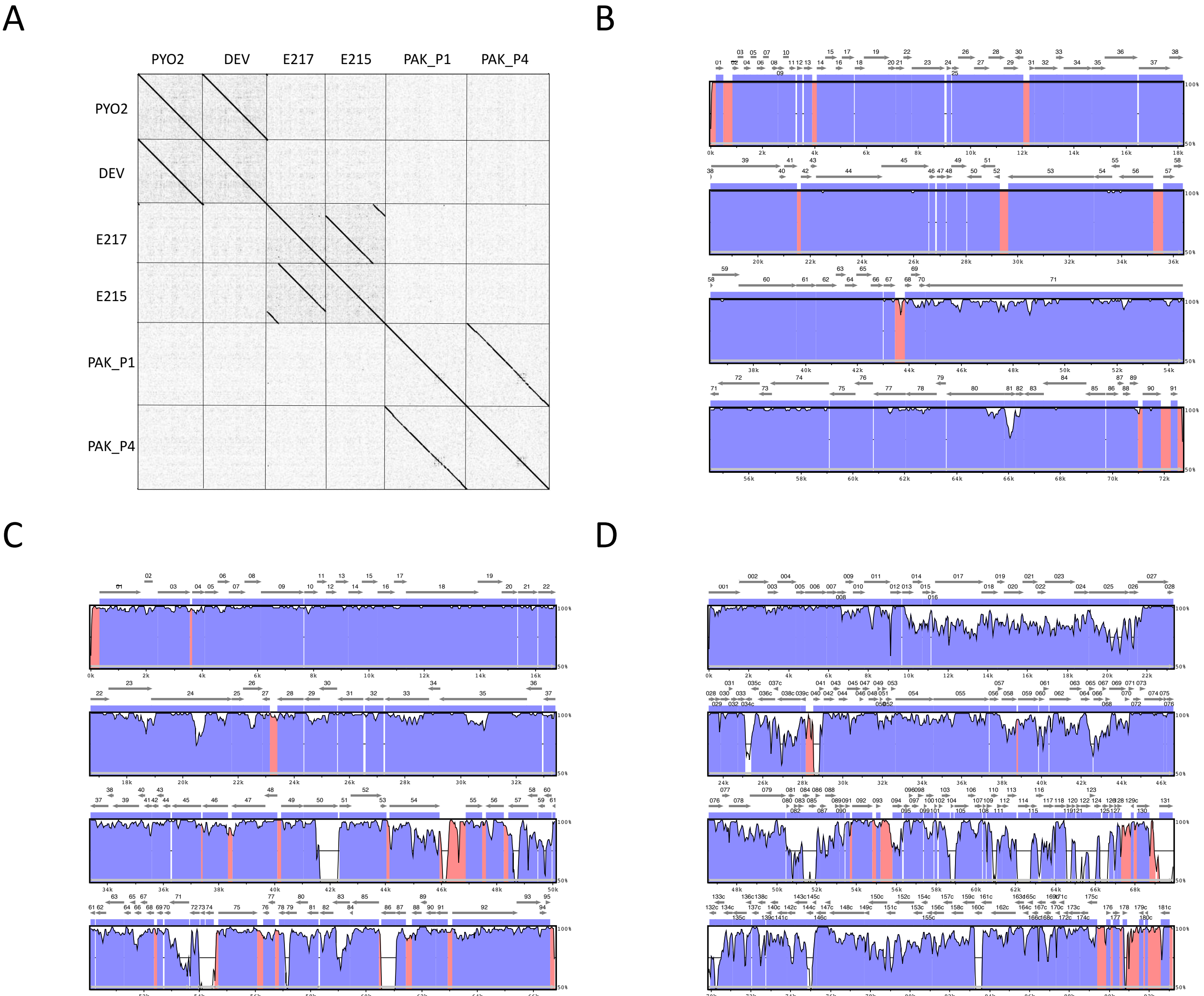

A
B
C
D
PYO2
DEV
E217
E215
PAK_P1
PAK_P4

Figure S4. Comparison between phage genomes. (A) Dot plot showing patterns of colinearity and identity between genomes employed in final cocktail. All pairwise comparisons of genome sequences are shown. The continuous diagonal shows self-identity for each genome. PYO2 and DEV, E215 and E217 as well as PAK_P1 and PAK_P4 each show high levels of identity in pairwise comparison, while no similarity is evident in other comparisons [word-length = 10, figure prepared using GEPARD (1)]. **(B-D)** Schematics showing patterns of sequence similarity in aligned genomes: B: DEV/PYO2 (DEV shown); C: E215/E217 (E215 shown); D: PAK_P1/PAK_P4 (PAK_P4 shown). In each case, dark grey arrows above the plots indicate the positions of predicted ORFs with numbers corresponding to the locus identifiers in the appropriate GenBank entries. Purple shaded regions indicate conserved coding sequences, red regions represent conserved non-coding sequences and pale grey arrows show the positions of contiguous aligned regions. Genome aligments and images prepared using wgVista (2).

REFERENCES

1. Krumsiek J, Arnold R, Rattei T. 2007. Gepard: a rapid and sensitive tool for creating dotplots on genome scale. Bioinformatics 23:1026-8.
2. Frazer KA, Pachter L, Poliakov A, Rubin EM, Dubchak I. 2004. VISTA: computational tools for comparative genomics. Nucleic Acids Res 32 (Web Server issue):W273-9.

Table S1. ***Pseudomonas aeruginosa*** **strains**

Laboratory strains

Strain	Relevant features	Origin or reference
PAO1	Laboratory strain	(1)
PAO1*pilA*	Deletion of the *pilA* gene	Imperi F.[a]

[a]Francesco Imperi, University of Rome-Sapienza, Italy.

Environmental isolates

Strain	Origin	Reference
E1	salad	(2)
E2	salad	(2)
E4	salad	(2)
E5	red pepper	(2)
E9	crème	(2)

Italian clinical strains

***P. aeruginosa* isolate**	**Patient**	**Disease**	**CFTR mutation**	***P. aeruginosa* status**	**Age**	**Origin**
CL1	CL	CF	F508del/F508del	intermittent	24	A
CL2	CL	CF	F508del/F508del	intermittent, mucoid	24	A
GS3	GS	CF	F508del/1717-1G>A	early infection	32	A
DV4	DV	COPD	----	chronic	26	A
AG5	AG	CF	F508del/2789+5G>A	chronic	49	A
AG6	AG	CF	F508del/2789+5G>A	chronic, mucoid	49	A
GA7	GA	CF	G542X/R1066H	chronic, mucoid	28	A
VR8	VR	CF	F508del/2183-AA>G	chronic	20	A
GJY9	GJY	CF	F508del/F508del	chronic	26	A
AA10	AA	COPD	----	chronic	9	A

PaPh1	OBG1	CF	F508del/G85E	1st infection	20	B
PaPh2	OBG2	CF	F508del/G542X	1st infection	22	B
PaPh3	OBG3	CF	1717-1G>A/S1455X	1st infection	1	B
PaPh4	OBG4	CF	F508del/G85E	1st infection	8	B
PaPh5	OBG5	CF	F508del/UN	1st infection	4	B
PaPh6	OBG6	CF	F508del/ F508del	1st infection	24	B
PaPh7	OBG7	CF	F508del/ Q685TfsX4	1st infection	5	B
PaPh8	OBG8	CF	F508del/ F508del	1st infection	38	B
PaPh9	OBG9	CF	F508del/ G542X	1st infection	15	B
PaPh10	OBG10	CF	F508del/ UN	early	23	B
PaPh11	OBG9	CF	F508del/ G542X	early	16	B
PaPh12	OBG1	CF	F508del/ G85E	early	21	B
PaPh13	OBG2	CF	F508del/ G542X	early	23	B
PaPh14	OBG4	CF	F508del/ G85E	early	9	B
PaPh15	OBG6	CF	F508del/ F508del	early	25	B
PaPh16	OBG11	CF	F508del/ L1077P	chronic	16	B
PaPh17	OBG11	CF	F508del/ L1077P	chronic	16	B
PaPh18	OBG12	CF	F508del/ 1259insA	chronic	34	B
PaPh19	OBG12	CF	F508del/ 1259insA	chronic	34	B
PaPh20	OBG13	CF	F508del/ N1303K	chronic	28	B
PaPh21	OBG14	CF	F508del/ F508del	chronic	38	B
PaPh23	OBG15	CF	F508del/ W128X	chronic	25	B
PaPh24	OBG16	CF	G542X/N1303K	chronic, MDR	20	B
PaPh25	OBG16	CF	G542X/N1303K	chronic, MDR	20	B
PaPh26	OBG12	CF	F508del/ 1259insA	chronic, mucoid	34	B
PaPh27	OBG14	CF	F508del/ F508del	chronic, mucoid	39	B
PaPh28	OBG17	CF	F508del/ 1717-1G>A	chronic, mucoid	29	B
PaPh29	OBG10	CF	F508del/ UN	early, mucoid	23	B
PaPh30	OBG18	CF	F508del/ 2183AA>G	early, mucoid	26	B

PaPh31	OBG19	CF	F508del/ N1303K	early, mucoid	25	B
PaPh32	OBG16	CF	G542X/N1303K	mucoid, post transplant, MDR	20	B
PaPh33	OBG20	CF	F508del/P5L	1st infection?	19	B

CF: Cystic Fibrosis; COPD: Chronic Obstructive Pulmonary Disease; UN: unknown.

A=Centro di Riferimento per la Fibrosi Cistica della Regione Lombardia, Milan, Italy.

B= Ospedale Bambino Gesù, Rome, Italy.

Strains with the same color were collected from the same patient at different times.

The strains were molecularly characterized with PFGE and the different patient strains were genetically different. This is an investigation done routinely in the laboratories as part of the cross-infection prevention surveillance program. Naturally, the early and late strains of the same patient are isogenic, but they differ for numerous phenotypic traits acquired during the adaptive radiation process, characteristic of chronic colonization.

Other clinical strains

***P. aeruginosa* isolate**	**Patient**	**Disease**	**CFTR mutation**	***P. aeruginosa* status**	**Age**	**Origin**
AA2	AA	CF	F508del/F508del	early infection	4	(2)
AA43	AA	CF	F508del/F508del	chronic, mucoid	11	(2)
AA44	AA	CF	F508del/F508del	chronic	11	(2)
TR1	TR	CF	F508del/F508del	early infection	7	(2)
TR66	TR	CF	F508del/F508del	chronic	20	(2)
TR67	TR	CF	F508del/F508del	chronic, mucoid	21	(2)

**Strain**	**Relevant features**	**Reference**
PA14	Clinical origin	(3)[c]
PAK-lumi	Bioluminescent; clinical origin non CF	(4,5)
LESB58	Clinical origin, CF	(6)[d]

[c]Received from Giovanni Bertoni, University of Milan, Italy.

[d]Received from Alessandra Bragonzi, Centro di Riferimento per la Fibrosi Cistica della Regione Lombardia, Milan, Italy.

REFERENCES

1. Stover CK, Pham XQ, Erwin AL, Mizoguchi SD, Warrener P, Hickey MJ, Brinkman FS, Hufnagle WO, Kowalik DJ, Lagrou M, Garber RL, Goltry L, Tolentino E, Westbrock-Wadman S, Yuan Y, Brody LL, Coulter SN, Folger KR, Kas A, Larbig K, Lim R, Smith K, Spencer D, Wong GK, Wu Z, Paulsen IT, Reizer J, Saier MH, Hancock RE, Lory S, Olson MV. 2000. Complete genome sequence of *Pseudomonas aeruginosa* PAO1, an opportunistic pathogen. Nature 406:959-64.
2. Bragonzi A, Paroni M, Nonis A, Cramer N, Montanari S, Rejman J, Di Serio C, Döring G, Tümmler B. 2009. *Pseudomonas aeruginosa* microevolution during cystic fibrosis lung infection establishes clones with adapted virulence. Am J Respir Crit Care Med 180:138-45.
3. Rahme LG, Stevens EJ, Wolfort SF, Shao J, Tompkins RG, Ausubel FM. 1995. Common virulence factors for bacterial pathogenicity in plants and animals. Science, 268:1899-1902.
4. Moir DT, Ming Di, Opperman T, Schweizer HP, Bowlin TL. 2007. A high-throughput, homogeneous, bioluminescent assay for Pseudomonas aeruginosa gyrase inhibitors and other DNA-damaging agents. J Biomol Screen 12:855-64.
5. Ramphal R, Balloy V, Jyot J, Verma A, Si-Tahar M, Chignard M. 2008. Control of *Pseudomonas aeruginosa* in the lung requires the recognition of either lipopolysaccharide or flagellin. J Immunol 181:586-92.
6. Cheng K, Smyth RL, Govan JR, Doherty C, Winstanley C, Denning N, Heaf DP, van Saene H, Hart CA. 1996. Spread of beta-lactam-resistant *Pseudomonas aeruginosa* in a cystic fibrosis clinic. Lancet 348:639-42.

Table S2. Phage collection.

NAME	ORIGIN[a]	HOST	PHAGE NOMENCLATURE	PHAGE ABBREVIATION	GENBANK ACCESSION NUMBER
2.	PHAGYO-Eliava	PAO1	vB_PaeP_PYO2	PYO2	MF490236
3.	INTESTIPHAGE-Eliava	PAO1			
DV3	3. mutant able to grow on DV4	PAO1			
C3.1	3. mutant large plaque	PAO1			
DV-CC	derived from DV3, plaques without halos	PAO1			
7 C3.1	C3.1 mutant able to grow on GA7	PAO1			
DV3-EV	evolution of DV3 in LESB58	PAO1	vB_PaeP_DEV	DEV	MF490238
E215-AG5a	E215	AG5	vB_PaeM_E215	E215	MF490241
E215-GS3b	E215	GS3			
B213-PA14d	B213	PA14			
E220-PAO1A	E220	PAO1			
E220-PA14A	E220	PA14	vB_PaeP_E220	E220	MF490237
E220-GS3B	E220	GS3			
S216-VR8	S216	VR8			
S218-GS3A	S218	GS3	vB_PaeS_E218	E218	MF490239
S218-GS3B	S218	GS3			
S220-AG5	S220	AG5			
S220-PAO1B	S220	PAO1			
B213a-AG5	B213	AG5			
E215-AG5	E215	AG5			
E217a-AG5	E217	AG5	vB_PaeM_E217	E217	MF490240
E219a-PA14	E219	PA14			
E1-b	spontaneous phage isolated on strain E1	E1			
PAK_P1	sewage water Paris	PAK	vB_PaeM_PAK_P1	PAK_P1	KC862297
PAK_P4	sewage water Paris	PAK	vB_PaeM_PAK_P4	PAK_P4	KC862300

[a]Phage isolated from sewage samples from: (E) Nosedo East; (S) Nosedo South; (B) Peschiera Borromeo. The numbers refer to samples collected in different days.

Table S3. Efficiency of plating on *P. aeruginosa* strains.

	EFFICIENCY OF PLATING OF																								
STRAIN	2./PYO2	3.	DV3	C3.1	DV-CC	7C3.1	E215-AG5a/E215	E215-GS3b	B213-PA14d	E220-PAO1A	E220-PA14A/E220	E220-GS3B	S216-VR8	S218-GS3A/S218	S218-GS3B	S220-AG5	S220-PAO1B	E1b	B213a-AG5	E215-AG5	E217-AG5/E217	E219a-PA14	DV3-EV/DEV	PAK_P1	PAK_P4
PAO1	+	+	+	+	+	+	+	+/-	-/+	+	+T	-/+	-	+/-	+	+	+T	+	+	+T	+	-	+	-	-
PA14	+/-	+	-	-	-	-	-	-	+	-	+	-	-/+	-	-	-	-	+	-	-	-	+	-	-	-
PAK-lumi	+	NT	NT	NT	NT	NT	+	NT	NT	NT	NT	NT	NT	NT	NT	NT	NT	NT	NT	NT	+	NT	+	+	+
PAO1 *pilA*	+	NT	NT	NT	NT	NT	+	NT	NT	NT	NT	NT	NT	NT	NT	NT	NT	NT	NT	NT	+	NT	+	-	-
LESB58	-	-	+/-	-	-/+	-	-	-	-	-	-	-	-	-	-	-	-	-	-	-	-	-	+	-	-
E1	+/-	+	-/+	-	-	-	+	-	-	-	-	-	-	-	-	-/+	-	+	+/-	-	+	-	-	-	-
E2	+	-/+	+	+	+	-	-	-	-	-	+T	-	-	-	-	+/-	+TT	-	-	+	-	-	+	-/+	-
E4	-	-	-	-	-	-	-	-/+	-	-	+/-T	-	-	-	-/+	-	-	-	+/-	-	-	-	-	-	-
E5	-	-	-	-	-	-	-/+	-	-	-	-	-	-	-	-	-	+T	-	-	-	-	-	-	-/+	-/+
E9	-	-	-	-	-	-	-	+	-/+	-	+/-	-	-/+	-	+	-	-	-	-	-	-	-	-	-/+	+/-
AG5	+	+	+	+	+	+	+	-	-	-	-	-	-	+/-	-	+	-	+	+	+	+	-	+	-/+	+/-
GS3	+	+	+	+	+	+	+	+	+/-	-	+	+	+	+	+T	+/-	+	+/-	+/-	-	+/-	-	+	+	+
AA10	+	+	+	+	+	+	-	-	-	-	-	-	-	-	-	-	-	-	-	+/-	-	-	+	-	-
GJY9	-	-	-	-	-	-	-/+	-	-	-	-	-	-	-/+	-	-	-	-	-	-	-	-	+/-	-	-
CL1	-	-	-	-	-	-	+/-	-/+	-	-	+/-T	-	+	-/+	-	-	-	-	-	-	-	-	+	-	-
CL2	-	-	-	-	-	-	+/-	+/-	-	-	+/-T	-	+	-/+	-/+T	+/-	-/+	-	+	-	+	-	-	-/+	-
VR8	+	+	+	+	+	+	+	+/-	+/-	-	+	-	+	+/-	+T	+	-/+	+	+	+	+	-	+/-	+	-
AG6	-	+T	-/+	-	-/+	-	+	-	-	-	-	-	-	-	-	-/+	-	-	-	-	-	-	-	-	-

DV4	+	-	+	+	+	-	-	-	-	-	-	-	-	-	-	-	-	-	-	-	-	-	+	-/+	+/-
GA7	+	+	-	-/+	-/+	+/-	-/+	-	-	-	-	-	-	+/-	-	+	-	+	+T	+	+T	-	-	+	+
AA2	+/-	-	-/+	+/-	+	-/+	-/+	-	-	-	+	-	+/-	-	-	?	+	-/+	-/+	-	-/+	-	-	-	-/+
AA43	+	+	+	+	+	-/+	+	-	-	-/+	+	-	+/-	-	-	+	+	+	+	-/+	+	-	+/-	-	-
AA44	+	+	+/-	+/-	+	-/+	+/-	-	-	-	+	-	+/-	-	-	+	+	+	+	-	+	-	-/+	-	-
TR1	+	-/+	+	+	+	+/-	-	-/+	-	-	-	-	-	-	-	-	-	-	-	+	-/+	-	+	-	-
TR66	+	-/+	+	+	+	-/+	-	-	-	-	-	-	-	-	-	-	-	-	-	-	-	-	+/-	-	-
TR67	+	-	+	+	+	+	-	-	-	-	-	-	-	-	-	-	-	-	-	-	-	-	+	-	-
PaPh1	+/-T	-	+/-T	+/-T	+/-T	-	-	-	-	NT	-	NT	-	-	-	-	-	-	-	-	-	NT	+	-	-
PaPh2	-	-	-	-	-	-	-	-	-	NT	-	NT	-	+/-	-	+/-	-	-	+/-	-	+/-	NT	-	-	-
PaPh3	+	-	+	+	+	+/-	-	-	-	NT	+/-	NT	-	-/+	+/-T	-	-	-	-/+	+	-/+	NT	+	+/-	-/+
PaPh4	+	+	+	+	+	+	+	-	-	NT	-/+	NT	-	-	+	+	-/+	+	+	+	+	NT	+	-	-
PaPh5	+	+	+	+	+	+	-/+	+	-	NT	+	NT	+	-/+	+T	+	+	+	+	+	+	NT	+	+	+/-
PaPh6	-/+	-	-	-	-	-	-	-	-	NT	-	NT	-	-	-	-/+	-/+	-	-	-	+	NT	-	-	-
PaPh7	-	+	-	-	-	-	+	-	-	NT	-	NT	-	-	-	+	-/+	+/-	+	-/+	+	NT	-	-	-
PaPh8	+T	-/+	+	+	+	-	-	-	-	NT	-	NT	-	-	-	-	-	-	-	-	-	NT	+	+/-	+/-
PaPh9	-	+	-/+	-	-/+	-	+	-	-	NT	-	NT	+/-	-/+	-/+	+	-/+	+	+	-	+	NT	-	-	-
PaPh10	-	-	-	-	-	-	-	+/-	-	NT	+/-	NT	-	-	+	-	-	-	-	-	-	NT	-	-	-
PaPh11	-	+	-	-	-	-	+	-/+	-	NT	-/+	NT	+/-	-	-/+	+	-	+/-	+	-	+	NT	-	-	-
PaPh12	+	-	+/-T	+T	-/+	-	+	-	-	NT	-	NT	-	-	-	+/-	-	-	-	-	-/+T	NT	+/-T	-	-/+
PaPh13	-	-	-	-	-	-	-/+	-	-	NT	-	NT	-	-/+	-/+	-/+	-	-	-/+	-	-/+	NT	-	-	-
PaPh14	+	+	+	+	+	+/-	+	-	-	NT	-	NT	-	-/+	-/+	+	-/+	+	+	+	+	NT	+	-	-
PaPh15	-/+	-	-/+	-	-	-	-	-	-	NT	-	NT	-	-	-	-	-	-	-	-	-	NT	+/-T	-	-
PaPh16	+	+	+	+	+	+	+	-	-	NT	-/+	NT	-	-	-	+	-/+	+	+	-/+	+	NT	+	-	-
PaPh17	+	+	+	+	+	+	+	-	-	NT	-	NT	-	-/+	-/+	+	-/+	+/-	+	-/+	+	NT	+	-	-
PaPh18	-	-	-	-	-	-	-	-	-	NT	-	NT	-	-	-	-	-	-	-	-	-	NT	-/+	-	-
PaPh19	-	-	+/-T	-	-	-	-	-	-	NT	-	NT	-	-	-	-	-	-	-	-	-	NT	+T	-	-
PaPh20	-	+/-	-	-	-	-	+/-	-	-	NT	-	NT	-	-	-	-	-	+/-	-	-	-	NT	-	-	-
PaPh21	-	-	-	-	-	-	+	-	-	NT	-	NT	-	-	-	-/+	-	-	-/+	-	-/+	NT	-	-	-
PaPh23	-	-	-	-	-	-	-	-	-	-	-	-	-	-	-	-	-	-	-	-	-	-	-	-	-
PaPh24	+	-/+	+	+	+	-	-	-	-	NT	-	NT	+	-	-	+/-	-	-	+/-	-	+/-	NT	+	+	+/-

PaPh25	+/-	+	+/-	+/-	+/-	+	-	-	-/+	NT	-	NT	+	+	-	+	-	-	-	+/-	+	NT	+/-	+/-	-/+
PaPh26	-/+	-	+/-	+/-T	+/-T	-	-	-	-	NT	-	NT	-	-	-	-	-	-	-	-	-	NT	+	-	-
PaPh27	-	+/-	-	-	-	-	+	-	-	NT	-	NT	-	-	-	+	-/+	+/-	+/-	-	+	NT	-	-	-
PaPh28	-/+	+	-	-	-	-	+	-	-	NT	-/+	NT	-	-	+/-	+	-/+	+	+	-	+	NT	-	-	-
PaPh29	-/+	-	-	-	-	-	-	+/-	-	NT	+	NT	-/+	-	-/+	-	-	-	-	-	-	NT	-	-	-
PaPh30	-	-	-	-	-	-	-	-	-	-	-	-	-	-	-	-	-	-	-	-	-	-	-	-	-
PaPh31	-	-	-	-	-	-	-/+	-	-	NT	-	NT	-	-	-	-	-	-	-	-	-/+	NT	-	-	-
PaPh32	-/+	-/+	-/+	-/+	-/+	-	+	-	-	NT	-	NT	-	-	-	+	-	-/+	+/-	-/+	+	NT	-/+	+/-	+/-
PaPh33	+	-	+	+	+	+	-/+	+T	-	NT	-/+	NT	-	-/+	-/+	-	-	-	-	+/-	-	NT	+	+/-	-

(+)	25	21	20	21	22	12	21	4	1	1	10	1	7	2	7	18	8	14	16	10	23	1	24	6	3
(+/-)	5	2	7	5	3	4	4	5	2	0	6	0	5	5	2	6	0	6	7	3	3	0	7	5	7
(-/+)	6	6	6	2	6	4	8	4	3	1	5	1	3	10	9	5	11	2	4	5	7	0	3	6	5
(-)	22	27	23	28	25	36	25	43	50	24	35	24	41	39	38	27	37	34	29	38	25	25	24	41	43
NT	0	2	2	2	2	2	0	2	2	32	2	32	2	2	2	2	2	2	2	2	0	32	0	0	0
Total	58	58	58	58	58	58	58	58	58	58	58	58	58	58	58	58	58	58	58	58	58	58	58	58	58

Phages that were renamed are indicated with the original name followed after a slash by the new name.

The efficiency of plating of the isolated phages on the panel of 58 strains of our collection is reported. 5 μl of ten-fold serial dilutions were spotted on a lawn of each specific bacterial host; the plates were observed after o.n. incubation at 37°C.

(+) = eop 1; (+/-) = eop 10^{-1}-10^{-2}; (-/+) = eop 10^{-3}; (-) = eop <10^{-4}. T = turbid plaques. NT = not tested.

Table S4. Genome assembly.

PHAGE NAME	Accession number	Taxonomy	Length (nt)	Most similar to:<br>name, (accession), % query cover, (% identity)	Presence of putative integrase
vB_PaeP_PYO2	MF490236	Caudovirales; Podoviridae; Lit1virus	72697	Pseudomonas phage PEV2 (KU948710.1) 100% (99%)<br>Pseudomonas phage RWG (KM411958.1) 100% (99%)	No
vB_PaeP_DEV	MF490238	Caudovirales; Podoviridae; Lit1virus	72697	Pseudomonas phage PEV2 (KU948710.1) 100% (99%)<br>Pseudomonas phage RWG (KM411958.1) 100% (99%)	No
vB_PaeM_E215	MF490241	Caudovirales; Myoviridae; P1virus; unclassified Punalikevirus	66789	Pseudomonas phage vB_PaeM_CEB_DP1 (KR869157) 97% (97%)<br>Pseudomonas phage vB_PaeM_CEB_DP1 (KR869157) 97% (97%)	No
vB_PaeM_E217	MF490240	Caudovirales; Myoviridae; P1virus; unclassified Punalikevirus	66291	Pseudomonas phage vB_PaeM_PAO1_Ab27 (LN610579) 98% (97%)<br>Pseudomonas phage vB_PaeM_CEB_DP1 (KR869157) 99% (97%)	No
vB_PaeP_E220	MF490237	Caudovirales; Podoviridae	62874	Pseudomonas phage LKA5 (KC900378) 84% (98%)	Yes
vB_PaeS_S218	MF490239	Caudovirales; Siphoviridae	61680	Pseudomonas phage AN14 (KX198613.1) 96% (98%)	Yes

The genome sequences of the phages are deposited in GenBank.
